# Book Notices

**Published:** 1882-05-20

**Authors:** 


### *BOOK NOTICES.*

ILLUSTRATIONS OF DISSECTIONS IN A SERIES OF ORIGINAL COLORED PLATES THE SIZE OF LIFE: Representing the Dissection of the Human body. By George Viner Ellis, Prof. of Anatomy in University College, London, and G. H. Ford, Esqr. The drawings are from nature by Mr. Ford, from dissections by Prof. Ellis. Volume II, Second Edition. New York: William Wood & Co. McGarity & Laird, Agents, Atlanta, Ga.

This is the second volume of the valuable work reviewed in our April issue. What was said of the first volume may also be said of this one.

MATERIA MEDICA & THERAPEUTICS, INORGANIC SUBSTANCES. By Charles D. F. Phillips, M. D., Member of the Royal College of Physicians, etc.; Late Lecturer on Materia Medica and Therapeutics, at the Westminster Hospital Medical School. Edited and adapted to the United States Pharmacopœia, by Lawrence Johnson, A.M., M.D., Lecturer on Medical Botany, Medical Department of the University of the City of New York; Fellow of the New York Academy of Medicine, etc. Volume I. New York: William Wood & Co. McGarity & Laird, Agents, Atlanta, Ga., 1882.

This is the second part of Dr. Phillips' Materia Medica, the first being devoted to the Vegetable Kingdom, and issued several years ago. The work is eminently interesting and practical.

SUPPRESSION OF URINE, CLINICAL DESCRIPTIONS AND ANALYSES OF SYMPTOMS. By E. P. Fowler, M. D. Ninety-three Clinical Cases, with illustrations, tables and diagrams. New York: William Wood & Co. 1881; oc. 86 pages.

The above is an interesting work on the subject treated, being a paper presented to the New York Medical and Chirurgical Society, December, 1880.

---

A COLLEGE for Medical Practitioners has been inaugurated at St. Louis, Mo. Object: To teach practitioners, by practical instruction, the practical branches of Medicine and Surgery.

**North Carolina Pharmaceutical Association.**—The Second Annual Meeting of this Association will be held in New Berne, on August 9th. Mr. T. C. Smtth, of Charlotte. is Secretary, Mr. F. W. Hancock, of New Berne, will attend to all local business, such as taking charge of exhibits, etc.

The Governor of the State has appointed the following gentlemen as the Board of Pharmacy for the State of North Carolina: William Simpson, of Raliegh; W. H. Green, of Wilmington; A. S. Lee, of Raleigh; E. M. Nadal, of Wilson; E. H. Meadow, of New Berne.—*New Remedies.*

